# Reconciling Signal-Detection Models of Criterion Learning with the Generalized Matching Law

**DOI:** 10.1007/s42113-024-00212-w

**Published:** 2024-08-01

**Authors:** Christina Koß, Luis de la Cuesta-Ferrer, Maik C. Stüttgen, Frank Jäkel

**Affiliations:** 1 Centre for Cognitive Science, Institute of Psychology, Technical University of Darmstadt, Darmstadt, Germany; 2 Institute of Pathophysiology, University Medical Center of the Johannes Gutenberg University Mainz, Mainz, Germany

**Keywords:** Decision making, Signal detection theory, Matching law, Criterion learning model

## Abstract

To make decisions that lead to favorable outcomes, animals have to consider both their perceptual uncertainty as well as uncertainty about the outcomes of their actions, such as reinforcements. There is a long tradition of research investigating how the reinforcement structure of a task controls animals’ response behavior. The relation between reinforcement and response rates has been described by the matching law and its generalizations for tasks with and without perceptual uncertainty. The influence of perceptual uncertainty on decision behavior is traditionally modeled with signal detection theory, which posits that a decision criterion is placed on an internal evidence axis. Where this criterion is placed and how it is updated based on reinforcements are open questions within signal detection theory. Various criterion learning models have been proposed; however, their steady-state behavior across different experimental conditions is not consistent with the aforementioned empirical matching laws. Here, we integrate models of criterion learning from signal detection theory with matching laws from animal learning theory to gain a better understanding of the mechanisms by which reinforcements and perceptual uncertainty jointly shape behavior. To do so, we first derive the criterion position that leads to behavior aligned with those laws. We then develop a model that updates the decision criterion trial by trial to learn this criterion position. Our model fits data from a previous experiment well and generates behavior in simulations that is in line with matching laws for perceptual tasks and the subjects’ behavior in the experiment.

## Introduction

How do animals choose actions that lead to favorable outcomes? This question is central to understanding adaptive behavior and, ultimately, its neural underpinnings. In the real world, many decisions involve perceptual uncertainty as well as uncertainty about the outcomes of an action. Therefore, a significant amount of behavioral and neuroscientific research revolves around understanding these two aspects of perceptual decision making (Abbott et al., 2017; Heekeren et al., 2008; Hanks & Summerfield, 2017; Najafi & Churchland, 2018). The two most common experimental decision-making paradigms present animals with signal-detection problems (focusing on perceptual uncertainty) or two-armed bandit problems (focusing on action outcome uncertainty). These two paradigms are complementary. In a typical signal-detection paradigm, one of two hard-to-distinguish stimuli is randomly chosen to be presented on each trial, and animals are trained to respond with two different actions, contingent on the stimulus. If the responses, after learning, are assumed to be a deterministic function of the perception of the stimuli, this paradigm is well-suited to study the role of perceptual uncertainty in decision making (but see Stüttgen et al., 2011a). In comparison, in a typical two-armed bandit paradigm, there are also two response options, but the responses are not controlled by different stimuli but instead by differential reinforcements. This paradigm is well-suited to study how animals learn about the expected rewards for each action. Unsurprisingly, if one of the two responses is reinforced with a higher probability than the other, then this action will be taken more frequently. Also, if the reinforcement probabilities change, the animals will adapt their behavior accordingly.

The two paradigms—signal detection and bandit prob-lems—can be combined. For example, as in a pure detection task, animals might have to respond with two different actions to two hard-to-distinguish stimuli. But unlike pure detection tasks where feedback is deterministic, correct responses are only reinforced probabilistically and the probability of reinforcement might be different for each response option (see, e.g., Stüttgen et al., 2011b, 2013). A satisfactory behavioral model for such combined experiments should capture experimental manipulations of perceptual uncertainty, prior stimulus probabilities, and differential reinforcements. It should also account for learning curves and for serial dependencies between responses in subsequent trials. Currently, there is no model that has been systematically studied and validated with regard to all these aspects. We therefore propose a new model that integrates three separate lines of theorizing: signal detection theory (Green & Swets, 1988), Markovian learning processes (Norman, 1974), and the matching law with its generalizations (Herrnstein, 1961; Baum, 1974; Davison & McCarthy, 1988). The first deals with models of perceptual uncertainty, the second with models of trial-by-trial learning, and the third with the steady-state behavior after learning. By combining insights from all three approaches, we hope to make progress towards a standard model for perceptual decision making in animals (cf. Rahnev & Denison, 2018). In this paper, we explain in detail how the three approaches are related conceptually. Our main contribution is, however, to demonstrate that matching-law behavior, as observed empirically in tasks with perceptual uncertainty, can be generated by a trial-by-trial criterion learning model within a signal detection theory framework.

### Related Work

Behavior in signal detection tasks is usually modeled within signal detection theory (Green & Swets, 1988), which decomposes a subject’s performance into a measure of sensitivity—how well can they discriminate between the different stimuli—and a measure of response bias, the decision criterion. Traditionally, a subject’s sensitivity is of primary interest in these studies, and the decision criterion is only of interest in as far as it allows for a more precise measurement of sensitivity. In classical models, the criterion is assumed to be fixed, but there are numerous extensions that try to model serial dependencies and learning curves. This is often done through some mechanism that updates the criterion from trial to trial depending on the feedback that is obtained in each trial (e.g., Kac, 1962; Friedman et al., 1968; Dorfman & Biderman, 1971; Thomas, 1973; Treisman & Williams, 1984; Erev, 1998; Stüttgen et al., 2013). The theoretically best-developed models are Markovian learning processes (Norman, 1972, 1974) among which the Kac-Dorfman-Biderman (KDB) model is the most natural extension of traditional signal detection models (Kac, 1962; Dorfman & Biderman, 1971). These models have been developed in the context of human psychophysics and have rarely been applied to animal behavior (but see Stüttgen et al., 2013). While they are theoretically elegant, these models do not seem to be consistent with the steady-state behavior of animals (Stüttgen et al., 2024).

An animal’s steady-state behavior depends on the reinforcement rates for different responses, and this relationship is captured well by Herrnstein’s matching law (Herrnstein, 1961) and its generalization (Baum, 1974), the so-called generalized matching law. These laws provide experimentally well-supported descriptions of the average steady-state behavior of an animal in a two-armed bandit problem. Importantly, however, they do not describe trial-by-trial adaptations of behavior. These can be modeled by classical reinforcement learning algorithms (Sutton & Barto, 1998), but other ideas, like melioration, have also been explored (Herrnstein & Vaughan, 1980; Vaughan, 1981; Vaughan & Miller, 1984). Since these models are based exclusively on experiments with two-armed bandit paradigms, it is not immediately clear how to best incorporate perceptual uncertainty. Some attempts have been made to develop psychologically plausible reinforcement learning models that work in settings with perceptual uncertainty (Lak et al., 2017; Funamizu, 2021). However, these do not try to incorporate empirical steady-state behavior but instead aim for behavior that maximizes expected reinforcement, as is customary for reinforcement learning algorithms in computer science.

Fortunately, there is an extensive body of experimental and theoretical work on integrating signal detection theory and the generalized matching law (Davison & Tustin, 1978; McCarthy & Davison, 1979; Davison & McCarthy, 1988). The upshot is that empirically the generalized matching law also applies to the steady-state behavior in experimental situations with perceptual uncertainty, but does so for each stimulus separately. We will refer to this version of the generalized matching law as the Davison-Tustin (DT) law. However, while the DT law unifies signal detection theory with the generalized matching law, it does not provide a mechanism for trial-by-trial updates of the criterion. To close this gap and combine the explanatory power of two well-established yet largely distinct areas of behavioral work, here, we will propose a Markovian criterion learning process that is consistent with the DT law.

### Overview

In the following, we will first review some background material on signal detection theory and the generalized matching law in the remainder of the introduction. Readers who are experts in one or both of these areas may want to skip the respective subsections. Then, in Section “Matching withPerceptual Uncertainty: The Davison-Tustin Law,” we will review some of the fundamental work on the DT law and show how to connect it to signal detection theory. This is, again, done in detail to cater to readers coming from two different backgrounds, an animal learning background as well as a signal detection theory background. In Section “CriterionLearning Models for the Davison-Tustin Law,” we will adapt the elegant KDB models that have been developed within signal detection theory in a way that they become consistent with the DT law. This is the main contribution of our paper. By design, the resulting new model accounts for both perceptual uncertainty and steady-state matching-law behavior. Just like the KDB models, it updates the criterion based on the feedback in each trial and can thus model learning curves as well. We will then fit the model to experimental data from a recent experiment (Stüttgen et al., 2024) and present some model simulations in Section “Fit to Experimental Data” to demonstrate that the model does indeed produce the desired steady-state behavior. Finally, in the discussion, we compare our approach to other approaches that could be taken to unify signal detection theory with the generalized matching law. In particular, we discuss attempts to extend reinforcement learning models to incorporate perceptual uncertainty.

### Signal Detection Theory

We will focus on one of the most common tasks in perceptual decision-making experiments: A stimulus that belongs to one of two categories, e.g., high vs. low pitch or familiar vs. unfamiliar items, is presented. The task is to classify the presented stimulus, i.e., decide which of the two categories it belongs to. In signal detection theory, such a task is called a yes-no task but has also been called single-interval identification (cf. Wichmann & Jäkel, 2018; Stüttgen et al., 2011a). In animal learning theory, this paradigm is referred to as two-stimulus two-choice conditional discrimination (Stüttgen et al., 2024). Each decision will then have an outcome depending on the choice and the actual category of the stimulus. Usually, correct responses will be rewarded (either deterministically or probabilistically), and incorrect responses will be punished or have no effect. There can also be some other kind of feedback indicating what the correct response was, but here we will only consider experiments where feedback is only given in the form of rewards.

**Fig. 1:**
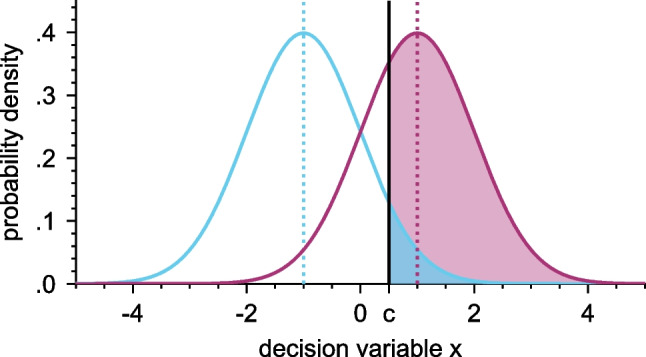
*Illustration of criterion setting in signal detection theory.* The two colored lines are the probability densities $p(x|S=i)$ for stimulus 1 (blue) and 2 (purple), respectively. In this example, these are Gaussian distributions with $d'=2$ and variance $\sigma ^2=1$. Dashed lines denote the means of the distributions. The filled areas are the hit rate $h = P(R=2|S=2)$ (purple) and false alarm rate $f = P(R=1|S=1)$ (blue) when placing the decision criterion at *c* (black line)

Decision making in such a task is usually modeled by signal detection theory (SDT). SDT assumes that each stimulus gives rise to sensory signals that are evaluated on an internal decision axis for the task at hand. For example, “pitch” signals are translated into evidence for the “high” or the “low” category, and “familiarity” signals are translated into evidence for the “old” or “new” category. Perception is uncertain: The same stimulus will lead to different sensory signals on each trial and, hence, to different values of the decision variable. This can be expressed as a distribution $p(x|S=i)$, where *x* is the decision variable and $S=i$ denotes that stimulus *i* was presented (see Fig. 1 for an example). Usually, the distribution of the decision variable for a given stimulus is assumed to be Gaussian, and in the simplest case, the distributions for the two stimuli are assumed to have equal variances.

The optimal decision strategy maximizes expected rewards, which means that in every trial, the response *i* is selected for which the likelihood $P(\text {reward}|R=i,x)$ that this will yield a reward is highest. If the decision variable is monotonically related to the likelihood ratio, this will be achieved by placing a criterion on the internal decision axis and consistently emitting one response when the decision variable is below that criterion and the other response when it is above that criterion. The position of the optimal criterion depends on the expected rewards for each correct and incorrect response and on the stimulus presentation probabilities.

Of course, it is also possible to base the decision on a different criterion. This will lead to lower expected overall rewards. Human and animal experiments on perceptual decision making have shown that the criterion is often not optimal. For example, in human experiments with unequal reward probabilities for the two response options, the criterion does not shift enough to be optimal (Maddox, 2002). When stimulus presentation probabilities are shifted away from 50%, the criterion is closer to optimal (Maddox, 2002), but often still does not shift enough (Green & Swets, 1988). In contrast, in animal experiments, unequal reward probabilities for the two response options can produce criterion shifts which are larger than would be optimal (Stüttgen et al., 2013). Within human psychophysics, the question of how and where people place their criterion is a long-standing open problem (Dusoir, 1983; Hautus et al., 2021). Hence, while SDT provides a useful framework for describing stationary decision behavior, it does not specify any mechanism of criterion updating, nor does it predict a subject’s response behavior in a given condition. Our goal is to close these gaps in the theory. We will show in Section “Criterion Placement” how the position of the decision criterion can be predicted using matching laws from animal learning theory, which we will then use in Section “Deriving a Model that is Compatiblewith the DT Law” to design a mechanism of criterion updating accordingly.

### The Generalized Matching Law

A long tradition of research with animals is concerned with the question of how rewards control behavior. Most of this work does not deal with decisions under perceptual uncertainty but rather only looks at response options that are distinguished by their expected outcomes. They typically use different variants of two-armed bandit paradigms. In many cases, animal behavior has been found to follow Herrnstein’s matching law, which says that the proportion of responses for one option is equal to the proportion of reward gained from that option (Herrnstein, 1961):

1 $$\begin{aligned} \frac{R_1}{R_1+R_2} = \frac{Rf_1}{Rf_1+Rf_2}, \end{aligned}$$

which can also be expressed as

2 $$\begin{aligned} \frac{R_2}{R_1} = \frac{Rf_2}{Rf_1}, \end{aligned}$$

where $R_i$ is the total number of trials in which response *i* was chosen and $Rf_i$ is the number of reinforced trials in which response *i* was chosen.

Several strategies have been suggested that animals could apply to end up with this kind of behavior, such as different kinds of reward maximizing strategies, or melioration (Herrnstein & Vaughan, 1980). While such a behavior can be optimal in certain circumstance (Kubanek, 2017; Sakai & Fukai, 2008), it is suboptimal in others (Vaughan, 1981; Vaughan & Miller, 1984). Frequently, systematic deviations from the matching law are found, and behavior is better described by the so-called generalized matching law (Baum, 1974):

3 $$\begin{aligned} \frac{R_2}{R_1} = b \left( \frac{Rf_2}{Rf_1} \right) ^ a \end{aligned}$$

or conveniently expressed in logarithmic form as

4 $$\begin{aligned} \log \left( \frac{R_2}{R_1} \right) = a \log \left( \frac{Rf_2}{Rf_1} \right) + \log b. \end{aligned}$$

This equation includes a bias *b* towards one of the responses and a sensitivity to reward *a*. Behavior with $a<1$ is called *undermatching* and with $a>1$ *overmatching*.

**Fig. 2:**
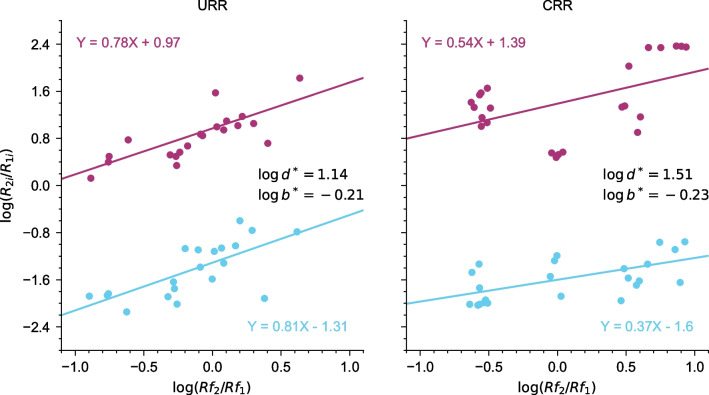
Reproduced from McCarthy and Davison (1979); the data were extracted with the help of WebPlotDigitizer (Rohatgi, 2021) from the plots for subject 123 in their Figs. 2 and 7. Left: data from an experiment with URR schedule, in which stimulus presentation probabilities were varied between conditions. Right: data from an experiment with CRR schedule, in which reinforcement ratio was varied between conditions. The logarithm of the response ratio $R_{2i}/R_{1i}$ is plotted as a function of the logarithm of the reinforcement ratio $Rf_{2}/Rf_{1}$ for the stimulus 1 (blue) and stimulus 2 (purple) trials. The best-fitting straight line and its equation are shown for each stimulus. The sensitivity $a_i$ in the DT law is directly given by the slope of the line for stimulus *i*; discriminability $\log d^*$ and bias $\log b^*$ were computed from $a_i$ and the lines’ intercepts

## Matching with Perceptual Uncertainty: The Davison-Tustin Law

The generalized matching law is not directly applicable to signal detection tasks since the original experiments do not address situations with perceptual uncertainty. However, building on a seminal theoretical paper by Davison and Tustin (1978), an extensive research program has investigated such scenarios since the late 70s. These authors hypothesized the following relation:

5 $$\begin{aligned} \frac{R_{21}}{R_{11}}&= \left( \frac{Rf_2}{Rf_1} \right) ^{a_1} b \frac{1}{d} \end{aligned}$$

6 $$\begin{aligned} \frac{R_{22}}{R_{12}}&= \left( \frac{Rf_2}{Rf_1} \right) ^{a_2} b d \end{aligned}$$

or in log-form

7 $$\begin{aligned} \log \left( \frac{R_{21}}{R_{11}} \right)&= a_1 \log \left( \frac{Rf_2}{Rf_1} \right) + \log b - \log d \end{aligned}$$

8 $$\begin{aligned} \log \left( \frac{R_{22}}{R_{12}} \right)&= a_2 \log \left( \frac{Rf_2}{Rf_1} \right) + \log b + \log d. \end{aligned}$$

$R_{ij}$ denotes the number of trials in which stimulus *j* was presented and response option *i* was chosen, and $Rf_i$ as before denotes the number of reinforced trials in which response option *i* was chosen.

For each stimulus, this is basically the generalized matching law but with an additional term *d* that shifts the response ratio towards response 2 for stimulus 2 and away from response 2 for stimulus 1. The value of *d* increases the more distinguishable the two stimuli are, which is why Davison and Tustin call this term *discriminability*.

We show in Appendix A that with these measures *b* and *d*, bias and discriminability are confounded in some situations. Hence, we define alternative measures of bias ($b^*$) and discriminability ($d^*$), such that Eqs. 7 and 8 become

9 $$\begin{aligned} \log \left( \frac{R_{21}}{R_{11}} \right)&= a_1 \log \left( \frac{Rf_2}{Rf_1} \right) + a_1 \log b^* - \log d^* \end{aligned}$$

10 $$\begin{aligned} \log \left( \frac{R_{22}}{R_{12}} \right)&= a_2 \log \left( \frac{Rf_2}{Rf_1} \right) + a_2 \log b^* + \log d^*. \end{aligned}$$

Importantly, however, this is simply a reparametrization of the same equations, substituting $\log b = (a_1+a_2)/2 \log b^*$ and $\log d = \log d^* + (a_2-a_1)/2 \log b^*$, and, therefore, does not change the empirical validity of the law. We refer to the empirical relations that are captured in these linear equations as the Davison-Tustin (DT) law. McCarthy and Davison (1979) tested the DT law using experimental schedules where the ratio of received reinforcements for either response option is under control of the experimenter (“controlled reinforcer ratio,” CRR) as well as schedules where this ratio can vary depending on the subject’s behavior (“uncontrolled reinforcer ratio,” URR).

A URR schedule can be implemented by presenting the two stimuli with probabilities $\pi _1$ and $\pi _2$, respectively, and giving the subject a reinforcement for a correct response to stimulus *i* with a fixed probability $r_i$ (“reinforcement rate”). The expected reinforcement that the subject receives for response *i* is then $Rf_i = \pi _i r_i P(R=i|S=i)$, where $P(R=i|S=i)$ denotes the probability of choosing response *i* given that stimulus *i* was presented. The experienced reinforcement ratio thus depends on the animal’s choices: choosing response *i* more often implies that more reward will be gained from response 1 than from response 2. The DT law fitted the data well in an experiment with a URR schedule, where the reinforcement rate for correct responses was held fixed while the stimulus presentation probabilities were varied between conditions (see Fig. 2, left).

To implement a CRR schedule, the reinforcement ratio $Rf_2/Rf_1$ for each experimental condition is chosen in advance and controlled by using two dependent variable interval schedules that provide reward availability for the two responses according to the chosen ratio. The subject’s response behavior can therefore not influence the experienced reinforcement ratio but only the overall frequency with which reinforcements are received. In their experiments with CRR schedule, McCarthy and Davison (1979) observed the following. When holding the reinforcement ratio fixed and varying stimulus presentation probability between conditions, the observed response ratio stays constant, which is also what the DT law predicts. When instead holding stimulus presentation probability fixed and varying reinforcement ratio between conditions, they observe that the behavior also follows the DT law (see Fig. 2, right). Across subjects, the fitted sensitivity to reinforcement *a* is notably lower in this CRR experiment compared to the URR experiment. This means that in the CRR procedure, the obtained reinforcement influenced animals’ behavior less, which is not too surprising considering that the animal has much less behavioral control over the rewards it obtains—in each trial, a reward can only be gained from one specific side.

The DT law has been further scrutinized in a series of studies. For an extensive review, see Davison and McCarthy (1988, Chapter 11). To summarize, they found that the model describes behavior well in scenarios with two distinct stimuli. Often, it is found that $a_1 = a_2$, i.e., the response ratios are equally susceptible to changes in income proportions for both stimuli. Similar to findings in scenarios without perceptual uncertainty, observers are often found to undermatch ($a<1$), i.e., the deviation of response ratio from 1 is less extreme than the deviation of reinforcement ratio from 1. Unfortunately, but also unsurprisingly, when category distributions become more complex and there are more than two stimuli, the law fails to accurately capture behavior (Davison & McCarthy, 1987).

### ROC Curves Implied by the DT Law

Our goal in this paper is to connect these well-established findings about animal behavior in perceptual decision-making tasks to signal detection theory in order to gain insight into how the steady-state decision criterion comes about. Traditionally in the matching-law literature, the connection between signal detection models and the DT law is framed in terms of Luce’s choice model (Davison & Tustin, 1978; Nevin et al., 1982), which is an alternative to the classical Gaussian signal detection models (Luce, 1963). Ignoring the choice-theoretic background of Luce’s model (Luce, 1959), here, we simply treat it as a standard signal detection model where the Gaussian distributions have been replaced by logistic distributions. We will show that the DT law implies logistic stimulus distributions for a signal detection model. Thereby, we also make the connection to Luce’s model, while explicitly working with a random variable representation, which is more customary to SDT and allows for placement of a decision criterion.

In the following, we will derive the logistic stimulus distributions via the receiver-operator characteristic (ROC), also called iso-sensitivity curve, which constitutes one of the most important concepts in SDT (Green & Swets, 1988; Hautus et al., 2021). It shows how an observer’s hit rate and false alarm rate relate to each other under varying experimental conditions. We can derive the ROC curve for an SDT model from the DT law as follows: Without loss of generality, we call the stimulus with lower mean stimulus 1 (“*noise*”) and the other one stimulus 2 (“*signal*”). The logarithmic response ratios in the DT law ($\log (R_{21}/R_{11})$ and $\log (R_{22}/R_{12})$) are then simply the log-odds of the false alarm rate $f=P(R=2|S=1)$ and hit rate $h=P(R=2|S=2)$, respectively, where the log-odds are defined as

11 $$\begin{aligned} \sigma ^{-1}(p) = \log \left( \frac{p}{1-p} \right) \end{aligned}$$

with the inverse being the logistic function

12 $$\begin{aligned} \sigma (x) = \frac{1}{1+e^{-x}}. \end{aligned}$$

Note that the logistic function $\sigma$ is the cumulative distribution function of the logistic distribution and that the inverse $\sigma ^{-1}$ is also called logit transform. The logit transform is frequently used as an alternative to the probit transform—the inverse $\Phi ^{-1}$ of the cumulative distribution function of the standard normal distribution $\Phi$—as a link function in generalized linear models. With this notation, we can replace the left-hand side of the DT law (Eqs. 9 and 10) with the logit transform and write the DT law equivalently as

13 $$\begin{aligned} \sigma ^{-1}(f)&= a_1 \log \left( \frac{Rf_2}{Rf_1} \right) + a_1 \log b^* - \log d^* \end{aligned}$$

14 $$\begin{aligned} \sigma ^{-1}(h)&= a_2 \log \left( \frac{Rf_2}{Rf_1} \right) + a_2 \log b^* + \log d^* \end{aligned}$$

We can now rearrange both equations in a way that the experimentally manipulated reinforcement ratios are on the right-hand sides of the equations:

15 $$\begin{aligned} \frac{1}{a_1} \left( \sigma ^{-1}(f) + \log d^* \right) - \log b^*&= \log \left( \frac{Rf_2}{Rf_1} \right) \end{aligned}$$

16 $$\begin{aligned} \frac{1}{a_2} \left( \sigma ^{-1}(h) - \log d^* \right) - \log b^*&= \log \left( \frac{Rf_2}{Rf_1} \right) . \end{aligned}$$

For both equations to hold simultaneously, the two left-hand sides need to be equal, which we can use to derive the ROC curve, i.e.  the hits as a function of the false alarms:

17 $$\begin{aligned} \frac{1}{a_1} \left( \sigma ^{-1}(f) + \log d^* \right) - \log b^*&= \frac{1}{a_2} \left( \sigma ^{-1}(h) - \log d^* \right) - \log b^* \end{aligned}$$

18 $$\begin{aligned} \Leftrightarrow \frac{1}{a_1} \sigma ^{-1}(f) + \frac{1}{a_1}\log d^*&= \frac{1}{a_2} \sigma ^{-1}(h) - \frac{1}{a_2}\log d^* \end{aligned}$$

19 $$\begin{aligned} \Leftrightarrow \sigma ^{-1}(h)&= \frac{a_2}{a_1} \sigma ^{-1}(f) + \left( 1+\frac{a_2}{a_1}\right) \log d^* . \end{aligned}$$

**Fig. 3:**
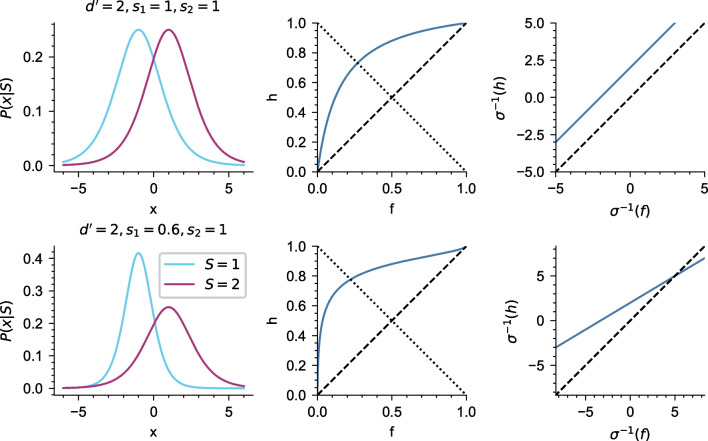
*SDT with logistic stimulus distributions.* Top row: example with equal variances for both stimuli, bottom row: example with unequal stimulus variances. Left column: stimulus distribution functions, middle column: ROC curve, right column: logit-transformed ROC curve

The relation between hits and false alarms is linear in logit space. This is inconsistent with the commonly used equal-variance Gaussian model of SDT that is linear in probit space. Instead, the DT law implies a signal detection model with an ROC curve (19) that results from logistic stimulus distributions. The logistic distribution is defined as

20 $$\begin{aligned} p(x|S=i) = \frac{e^{-\frac{x-\mu _i}{s_i}}}{1 + e^{-\frac{x-\mu _i}{s_i}}} =: \mathcal {L}\left( x; \mu _i, s_i \right) . \end{aligned}$$

with stimulus means $\mu _1=-d'/2$ and $\mu _2=d'/2$, where $d' = \left( 1+\frac{a_2}{a_1}\right) \log d^*$, and stimulus variances $s_1=a_2/a_1$ and $s_2=1$. Appendix B.1 gives a derivation that this distribution does indeed give rise to the same ROC curve as the DT law. Note also that—as always in SDT—the ROC curve stays the same if $s_1$, $s_2$, and $d'$ are scaled by a common factor, or both $\mu _1$ and $\mu _2$ are shifted by the same amount. For the symmetric case $a_1=a_2$, which is often found empirically, Eq. 19 simplifies to

21 $$\begin{aligned} \sigma ^{-1}(h) = \sigma ^{-1}(f) + d'. \end{aligned}$$

This corresponds to a symmetric ROC curve resulting from logistic stimulus distributions with $s_1=s_2=1$ and $d'=2 \log d^*$. This equation is analogous to the standard equation to compute $d'$ in the equal-variance Gaussian model: $d'=\Phi ^{-1}(h)-\Phi ^{-1}(f)$, which is why we have used the same symbol $d'$ to make this analogy more visible. But since in the equal-variance Gaussian model the probit transform $\Phi ^{-1}$ is used instead of the logit transform $\sigma ^{-1}$, the values for $d'$ will be different in the two models. Example stimulus distributions and the corresponding ROC curves for the logistic model are shown in Fig. 3. Qualitatively, there is no big difference between the equal-variance Gaussian model and the logistic model, and, in fact, both can be hard to distinguish empirically (Treisman & Faulkner, 1985).

### Criterion Placement

In the previous section, we eliminated the reinforcement ratio from the equations to derive the ROC curve, which describes all possible behavioral trade-offs, independent of the actually chosen behavior. This is in line with the traditional focus of signal detection theory that aims to characterize perceptual sensitivity rather than investigating how other factors, like the prior probabilities and the reward structure of the task, influence behavior (Green & Swets, 1988). But the DT law also directly describes how the obtained reinforcements influence behavior. Therefore, it does not only imply a logistic SDT model with corresponding ROC curves but, importantly, also predicts which criterion will be chosen in a specific experimental scenario. We call this the DT criterion.

For logistic stimulus distributions with means $\mu _1=-\frac{d'}{2}$, $\mu _2=\frac{d'}{2}$ and scales $s_1=\frac{a_2}{a_1}$, $s_2=1$, hit rate and false alarm rate are

22 $$\begin{aligned} h&= \sigma \left( -c+\frac{d'}{2} \right) \end{aligned}$$

23 $$\begin{aligned} f&= \sigma \left( \left( -c-\frac{d'}{2}\right) \frac{a_1}{a_2} \right) . \end{aligned}$$

Therefore, the criterion *c* can be computed from hit rate and false alarm rate as

24 $$\begin{aligned} c = - \frac{1}{2} \left[ \sigma ^{-1}(h) + \frac{a_2}{a_1} \sigma ^{-1}(f))\right] . \end{aligned}$$

According to the DT law, hit rate and false alarm rate directly depend on the reinforcement ratio. Plugging in Eqs. 13 and 14 for $\sigma ^{-1}(h)$ and $\sigma ^{-1}(f)$, after some algebra (shown in detail in Appendix B.2) yields

25 $$\begin{aligned} c = - a_2 \log \left( \frac{Rf_2}{Rf_1} b^* \right) + c_0 \end{aligned}$$

with

26 $$\begin{aligned} c_0 := - \frac{1}{2} \frac{a_1-a_2}{a_1+a_2} d', \end{aligned}$$

where $c_0$ is the neutral criterion, where the two stimulus distributions intersect (see Appendix B.3). Hence, the DT criterion can be decomposed into the neutral criterion plus a term that depends on the reinforcement ratio $Rf_2/Rf_1$ and the subject’s bias $b^*$. As $b^*$ shows up as a multiplicative factor to $Rf_2/Rf_1$, it can be interpreted as the subject behaving as if there was a reinforcement ratio $b^*$ already present when reinforcement is actually symmetric.

In a CRR schedule, $Rf_1 / Rf_2$ is held constant, so the DT criterion is directly given by Eq. 25. In a URR schedule, $Rf_1 / Rf_2$ depends not only on the programmed reinforcement rates but also on the subject’s response behavior—recall that $Rf_i = P(R=i|S=i)\pi _i r_i$ in such a schedule. Plugging this and the response probabilities in, we get

27 $$\begin{aligned} c = -a_2 \log \left( \frac{1+e^{-a_1/a_2 \left( d'/2+c\right) }}{1+e^{-\left( d'/2-c\right) }} \frac{\pi _2 r_2}{\pi _1 r_1} b^* \right) + c_0 \end{aligned}$$

(see Appendix B.2 for the details of the derivation). The criterion position is again the neutral criterion shifted by a bias resulting directly from the imbalanced condition and the inherent bias of the observer, but it also includes a recursive term that shifts the criterion further depending on how far it is shifted already. Conceptually, the reason for the recursion is that the criterion position depends on the ratio of received reinforcements, while the ratio of received reinforcements in turn also depends on the criterion position: the further shifted towards one side the criterion is, the less the observer will respond with that response option and therefore the less reinforcement they will receive from that side.

The recursive equation can be solved with numerical methods, e.g., fixed-point iteration, to compute the DT criterion for a condition with some $a_1$, $a_2$, $d^*$, and $b^*$. For $a_1<1$ and $a_2<1$, there is always a solution for any condition. A proof can be found in Appendix C. Hence, undermatching behavior according to the DT law is consistent with a logistic SDT model of decision making with a criterion at a fixed position.

## Criterion Learning Models for the Davison-Tustin Law

Now that we have interpreted the Davison-Tustin law in terms of signal detection theory and have found a description of the resulting criterion for different experimental conditions, we want to model the trial-by-trial learning process that ends up at this criterion position.

### The Kac-Dorfman-Biderman Model

A very natural way to implement a criterion learning model in signal detection theory has been developed by Dorfman and Biderman (1971) based on an idea by Kac (1962), which is why the resulting model is commonly referred to as the KDB model. The basic idea of this model is to update the criterion after each trial by a fixed amount based on feedback. The updates are chosen to make correct answers more likely and errors less likely. The criterion in trial $n+1$ is given by

28 $$\begin{aligned} c_{n+1}&= c_n + \Delta _{11}&\text { if }&R=1, \, S=1 \end{aligned}$$

29 $$\begin{aligned} c_{n+1}&= c_n + \Delta _{21}&\text { if }&R=2, \, S=1 \end{aligned}$$

30 $$\begin{aligned} c_{n+1}&= c_n - \Delta _{12}&\text { if }&R=1, \, S=2 \end{aligned}$$

31 $$\begin{aligned} c_{n+1}&= c_n - \Delta _{22}&\text { if }&R=2, \, S=2 \end{aligned}$$

The authors suggest different special cases as variants of the general model. For example, observers might update their criterion only after errors ($\Delta _{11} = \Delta _{22} = 0$) or only after correct responses ($\Delta _{12} = \Delta _{21} = 0$), or some of the update steps might be constrained to have the same size ($\Delta _{12}=\Delta _{21}$, $\Delta _{11}=\Delta _{22}$).

Several analyses have been carried out to investigate the asymptotic behavior of these and similar additive learning models (Norman, 1972; Thomas, 1973; Norman, 1974). A focus has been on error learning models, because there is a stationary distribution for the criterion (which is not necessarily the case for more general models that also learn on correct trials). Moreover, under some conditions, error learning models display probability matching behavior, i.e., the response probabilities match the stimulus probabilities. Probability matching has also been observed in humans doing tasks with feedback (Dorfman, 1969; Friedman et al., 1968). However, probability matching is not compatible with the DT law. The criterion that leads to probability matching for a certain condition only depends on the stimulus presentation probabilities and is independent of the reinforcement rates for each response option, while the DT criterion depends on both.

As the reinforcement ratios directly influence the response behavior according to the DT law, it is also more plausible to assume that animals learn from the reinforced trials rather than from their mistakes. However, models in which the criterion is updated only after correct responses are unstable, and there is no stationary distribution for the criterion; the criterion moves further and further outside, and the model eventually displays exclusive choice behavior. An extension of the KDB models that stabilizes reward learning has been suggested by Stüttgen et al. (2013). They introduce a leak term that prevents the criterion from diverging by pulling it back towards a neutral criterion at $c=0$. The update rule is given by

32 $$\begin{aligned} c_{n+1}&= \gamma c_n + \Delta _{11} &  \text { for rewarded } R=1 \text { trials} \end{aligned}$$

33 $$\begin{aligned} c_{n+1}&= \gamma c_n - \Delta _{22} &  \text { for rewarded } R=2 \text { trials} \end{aligned}$$

34 $$\begin{aligned} c_{n+1}&= \gamma c_n &  \text { for unrewarded trials } \end{aligned}$$

Note that in contrast to the original KDB model, the criterion in this model changes only after trials in which the subject receives a reward, not after every correct trial. This is to account for the situation that rewards might be given probabilistically rather than for every correct response, as it is often the case in animal experiments. The subjects in such experiments cannot clearly distinguish between correct but unrewarded responses and incorrect responses due to incomplete feedback. Their model was found to adequately fit adaptive choice behavior in some scenarios (Stüttgen et al., 2013), but has been less successful in others (Stüttgen et al., 2024). Moreover, this model’s asymptotic behavior is also incompatible with the DT law (see Appendix G for an explanation).

### Deriving a Model that is Compatible with the DT Law

Our approach here is to explicitly design a criterion learning model that is compatible with the DT law. This compatibility is a necessary property for any model of perceptual decision-making. The DT law does, for example, fit the data of McCarthy and Davison (1979), which are shown in Fig. 2. The validity of the DT law has been confirmed in many subsequent studies and can describe a wide range of data (Davison & McCarthy, 1988). In our own work, we have, not surprisingly, also found that animals’ behavior adheres to the DT law in the steady state (Stüttgen et al., 2024). In Section “Criterion Placement,” we derived the criterion which is in accordance with the DT law. We now develop a model with reinforcement-based learning that converges to this criterion. As we have seen, previously suggested criterion learning models with fixed update step sizes do not have this property and are therefore not consistent with the empirical findings from animal experiments. Therefore, the KDB reward learning model requires modification such that, instead of having steps of constant size, the size of the update steps $\Delta _{11}$ and $\Delta _{22}$ depends on the current criterion position. In the following, we derive which dependence $\Delta _{11}(c)$ and $\Delta _{22}(c)$ is needed for the model to converge to the DT criterion.

In criterion updating models, like the KDB models, the criterion is updated stochastically, because the update depends on the presented stimulus, the given response and the received reinforcement, which all are probabilistic. As long as the step sizes do not decrease over time, the criterion does not actually converge, but instead the model will asymptotically approach a steady state, in which the criterion keeps fluctuating around a certain numerical value. Showing that a steady-state criterion distribution exists and deriving it is beyond the scope of this paper (but see Norman (1974) for an analysis of the KDB model). However, we can derive the so-called *equilibrium criterion*, i.e., the criterion at which the expected update step is zero. Heuristically, the criterion does not change on average if at the current criterion position $\hat{c}$ the expected step in one direction is as big as the expected step in the other direction, i.e., the overall expected update step is zero:

35 $$\begin{aligned} \mathbb {E}(\Delta |\hat{c}) = P(R=1, \text {reward}|\hat{c}) \Delta _{11}(\hat{c}) - P(R=2, \text {reward}|\hat{c}) \Delta _{22}(\hat{c}) = 0. \end{aligned}$$

Recall that $Rf_i$ in the DT law denotes the expected number of reinforced $R=i$ trials, i.e., for a single trial

36 $$\begin{aligned} Rf_i(c) = P(R=i, \text {reward}|c). \end{aligned}$$

Using this, we can rewrite the equilibrium equation (35) as

37 $$\begin{aligned} \mathbb {E}(\Delta |\hat{c}) = Rf_1(\hat{c}) \Delta _{11}(\hat{c}) - Rf_2(\hat{c}) \Delta _{22}(\hat{c}) = 0 \end{aligned}$$

which is equivalent to

38 $$\begin{aligned} \frac{\Delta _{11}(\hat{c})}{\Delta _{22}(\hat{c})} = \frac{Rf_2(\hat{c})}{Rf_1(\hat{c})} \end{aligned}$$

We now derive a model whose behavior in the equilibrium fulfills the DT law by choosing the step-size functions $\Delta _{11}(\hat{c})$ and $\Delta _{22}(\hat{c})$ appropriately. As is usually done in SDT, we are assuming equal-variance stimulus distributions, i.e., $a_1=a_2=: a$. We also assume undermatching because this will ensure the existence of a criterion that is consistent with the DT law (see Appendix C). This is in line with the findings of Davison, Tustin, and McCarthy, who often observed $a_1\approx a_2<1$. A model will thus fulfill the DT law in the equilibrium if its equilibrium criterion is at

39 $$\begin{aligned}&\hat{c} = - a \log \left( \frac{Rf_2}{Rf_1} b^* \right) \end{aligned}$$

40 $$\begin{aligned} \Leftrightarrow&\frac{Rf_2}{Rf_1} = \frac{1}{b^*} e^{-\hat{c}/a} \end{aligned}$$

(see Eq. 25 in Section “Criterion Placement”). Plugging this into the equilibrium equation (38), we get

41 $$\begin{aligned} \frac{\Delta _{11}(\hat{c})}{\Delta _{22}(\hat{c})} = \frac{1}{b^*} e^{-\hat{c}/a}. \end{aligned}$$

A straight-forward way to get this to hold for the equilibrium criterion $\hat{c}$ is to choose $\Delta _{11}(c)$ and $\Delta _{22}(c)$ such that

42 $$\begin{aligned} \frac{\Delta _{11}(c)}{\Delta _{22}(c)} = \frac{1}{b^*} e^{-c/a}. \end{aligned}$$

for every *c*. For any model where the two step sizes have this ratio, the DT law holds in the equilibrium.

**Fig. 4:**
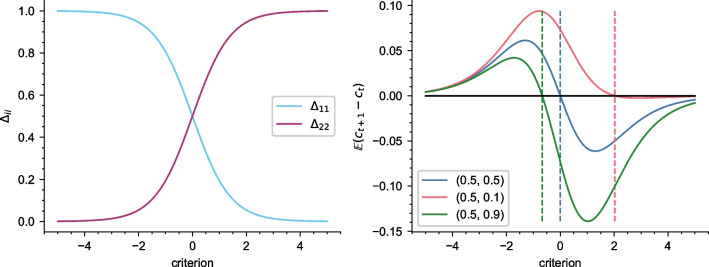
*Left:* Step-size functions $\Delta _{11}(c)$ and $\Delta _{22}(c)$ for $a=0.7$. *Right:* Expected update step dependent on the criterion position for different experimental conditions in a URR schedule (solid lines), and DT criteria for these conditions (dashed lines) for $a=0.7$ and $d'=2$. N.b. the DT criteria lie exactly at the zero-crossings of the expected update step, i.e., where the model is in an equilibrium. The slope of the curves is negative at the respective equilibrium positions, so the equilibrium is stable

There are many ways to choose step-size functions such that they have this ratio. To make our model more realistic, we impose the following additional constraints:

1. The step sizes should be symmetric, i.e., $\Delta _{11}(c)$ = $\Delta _{22}(-c)$.
2. They should be bounded, i.e., $\Delta _{ii}(c)\le \Delta _{\text {max}}$ for all *c*.

Assuming also an unbiased observer ($b^*=1$), these constraints lead us to choose the following step-size functions (see Appendix D for details):

43 $$\begin{aligned} \Delta _{11}(c)&= \Delta _\text {max} \frac{1}{1+e^{c/a}} = \Delta _\text {max} \frac{e^{-c/a}}{1+e^{-c/a}} \end{aligned}$$

44 $$\begin{aligned} \Delta _{22}(c)&= \Delta _\text {max} \frac{1}{1+e^{-c/a}} \end{aligned}$$

where $\Delta _\text {max}$ is a constant scaling parameter.

This, however, is not the only possible way to derive a step-size ratio that will asymptotically lead to behavior that conforms to the DT law. One might, for example, choose the step-size ratio to directly depend on the reinforcement ratio. This could be desirable because it seems plausible that animals adjust learning step sizes dependent on the amount of reinforcement they receive rather than only dependent on their own response behavior. An example derivation for step-size functions that depend on the received reinforcement is shown in Appendix E.

As in the original reward-based KDB model, the criterion in our model is updated such that a response that is reinforced becomes more likely subsequently. In a URR schedule, where the ratio between reinforcements for the two responses depends on the responses given, this mechanism has a self-reinforcing effect: A step taken in one direction makes it more likely to get reinforcement for that same response and thus to take another step in the same direction in the future. However, the model achieves stability by scaling the size of the steps. The further to one side the criterion is shifted, the more likely the model is to take another step in the same direction, but the smaller become the steps further into that direction and the larger the steps going back into the opposite direction (see Fig. 4, left). By design, these two forces balance out exactly at the criterion that gives rise to DT-law-consistent behavior (see Fig. 4, right). A proof that the model with these step-size functions has a unique and stable equilibrium for $a<1$ (undermatching, which is usually observed empirically—see, e.g., Baum (1979)) can be found in Appendix F.

## Fit to Experimental Data

**Fig. 5:**
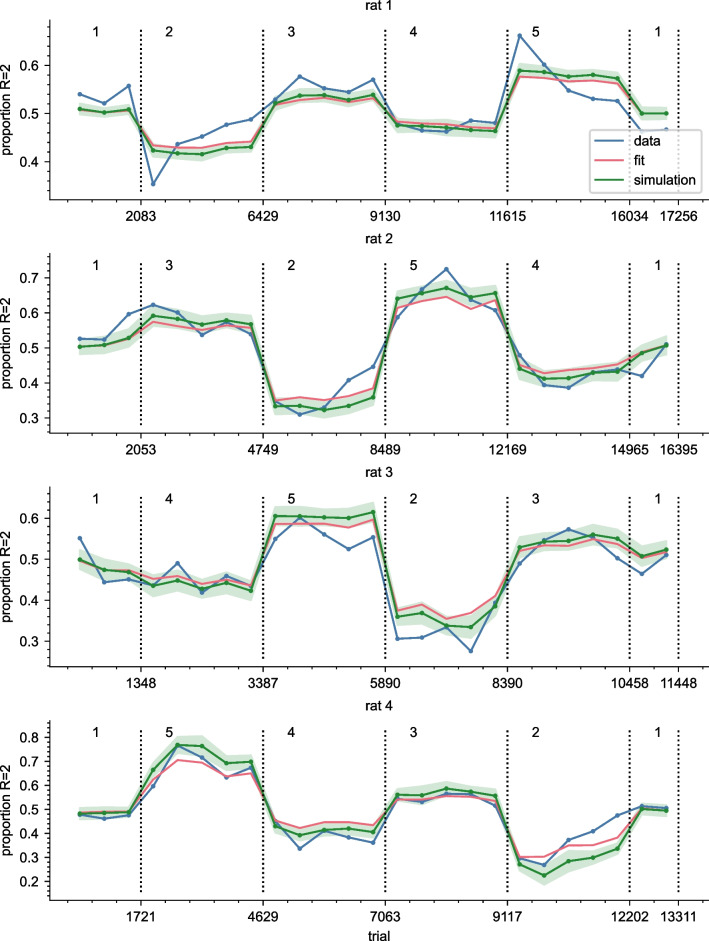
*Data, model fit, and simulations.* Each data point is the proportion of *R*=2 responses for one session. Blue: observed proportion for original data, red: predicted proportion for model fit, green: observed proportion averaged over 100 simulations, the shaded area indicates one standard deviation above and below the mean. Conditions end at the dotted black lines and are indicated by the numbers at the top of each plot. The reinforcement rates $r_1/r_2$ for each condition are 1 -.5/.5, 2 -.5/.1, 3 -.5/.9, 4 -.9/.5, 5 -.1/.5

We fit our model to existing data from experiment 1 described in Stüttgen et al. (2024). The data-set and the analysis code are available at the OSF project site https://osf.io/y8xek/. In the experiment, rats had to perform an auditory discrimination task where they had to distinguish between white noise bursts with two different center frequencies. The bandwidths of the stimuli were adjusted individually for each subject to yield 80% correct responses. In each trial, the subjects were presented with one of two possible stimuli that were presented with equal probability. They could respond by poking into one of two nose ports—the right one to indicate $S=1$ or the left one to indicate $S=2$. Correct responses were reinforced according to a URR schedule, i.e., there were fixed rates of reinforcement $r_1$ and $r_2$ with which correct $R=1$ or $R=2$ responses were reinforced. These reinforcement rates were varied between conditions (see Table 1), and the sequence of conditions was counterbalanced across subjects (see Fig. 5). Each experimental condition was run for 5 consecutive days. We refer to each of these as “sessions.” Additionally, a baseline condition with equal reinforcement rates for both responses was run for 3 sessions before and 2 sessions after the experimental conditions. A session lasted 45 minutes and contained a median of 551 trials. For more details on the subjects, stimuli, and procedure, see Stüttgen et al. (2024).

The model was fit by maximizing the log-likelihood of the data under the model. The parameters to be fitted are $\Delta _\text {max}$, $d^\prime$, and *a*. For a given $\Delta _\text {max}$ and *a*, the log-likelihood can be formulated as a generalized linear model with a unique maximum, which can therefore be maximized reliably using standard numerical optimization procedures (Dorfman, 1973). The optimal values for $\Delta _\text {max}$ and *a* were determined via grid search. For each combination of $\Delta _\text {max}$ (ranging from 0.001 to 2) and *a* (ranging from 0.1 to 1), the optimal $d^\prime$ and the corresponding log-likelihood were determined, and the parameters corresponding to the overall highest log-likelihood were chosen. The fitted parameter values for each subject can be found in Table 2. The results of the fit are visualized in Fig. 5, which shows the proportion of $R=2$ responses in each session for the original data along with the predictions from the fitted model.

**Table 1:** Experimental conditions in experiment 1 in Stüttgen et al. (2024)

Condition	$r_1$	$r_2$
1	0.5	0.5
2	0.5	0.1
3	0.5	0.9
4	0.9	0.5
5	0.1	0.5

Each condition follows a URR schedule in which correct response *i* trials are rewarded with probability $r_i$

To evaluate the goodness-of-fit, we compared our model to two existing models that our model was designed to improve upon: The income-based KDB model from Stüttgen et al. (2013) also updates the decision criterion after each received reinforcement, but does not take steady-state DT law behavior into account (see Section “The Kac-Dorfman-Biderman Model”). Stüttgen et al. (2024) made a first proposal for a trial-by-trial learning model that implements the DT law, but it uses a much less realistic update rule than our model. We fit each of the models to the same data-set (see Stüttgen et al., 2024, for details) and computed the Bayesian information criterion (BIC) for each fit (see Table 3). As expected, our model outperforms both of the previous models.

A good model fit does not necessarily imply though that the model would also generate behavior that is similar to the data. This is because the model predictions for each trial take into account the actual experimental history (i.e., stimuli, responses, and reinforcements) up to this trial, ignoring the possibility that the model might not be likely to have generated such a history (see Corrado et al. (2005) for an illustration). To check our model’s validity, we therefore also compared its generative behavior in the experiment to the actual subjects’ behavior. One hundred simulations of the model with the fitted parameters were run for each subject, using the original stimulus sequence the subject was confronted with and providing a reward for a correct response in the same trials that the subject originally could have gained one by responding correctly. The mean and standard deviation of the 100 model simulations are shown in Fig. 5, together with the original data and model fit. It can be seen that the model does indeed behave similarly to the subjects when faced with the same experiment sequence, although some of the data points lie several standard deviations away from the simulation mean. For example, the overshoot that subject 1 displays at the beginning of conditions 2 and 5 cannot be captured, as well as the asymmetry in response proportions between conditions 2 and 5 for subject 3.

**Fig. 6:**
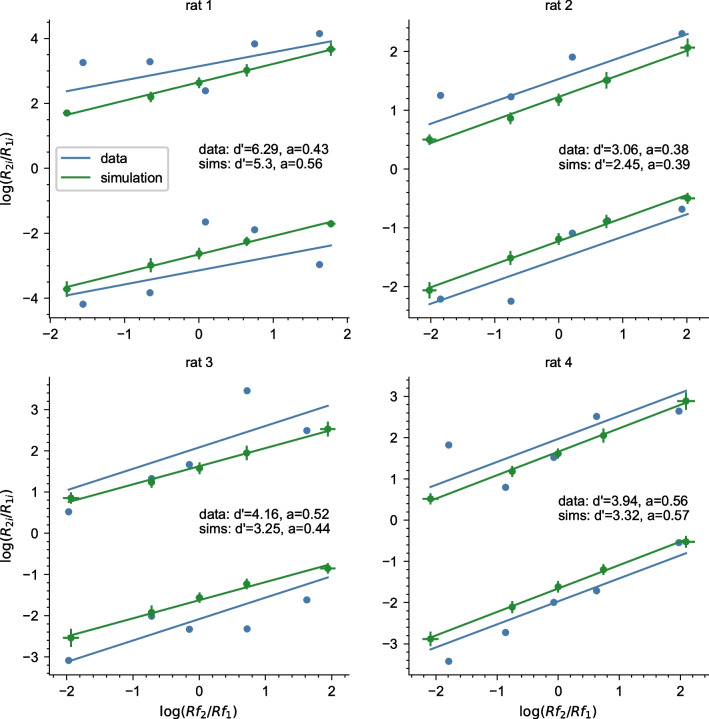
*Fitting the DT law.* For each experimental condition, the log response ratio is plotted against the log reinforcement ratio. Blue: original data, green: average of 100 simulations of the experiment, simulated with the parameters from the model fit on newly generated stimulus sequences. The horizontal and vertical bars indicate one standard deviation along each axis. Dots represent the data points, the lines result from fitting the DT law with $a_1=a_2 =: a$ and $\log b^* = 0$ to the data. The parameters of the DT-law fit (a, $d'=2 \log d^*$) are given in the plot

Next, we looked at the generative behavior of the model in the given experimental setup but independent of a specific experimental sequence, to validate that it does indeed conform to the DT law. To do so, another 100 model simulations per subject were run with the fitted parameters, this time newly generating stimulus sequence and potential reinforcements for each simulation, according to the same procedure that was used originally in the experiment in Stüttgen et al. (2024). Averaging over these simulations generates a prediction of how the model generally behaves in the experimental setup. To check whether the model’s behavior in the steady state conforms to the DT law, we computed response ratios and reinforcement ratios for each stimulus in the last two sessions of each condition and fitted the DT law with $a_1=a_2$ and $\log b^*=0$ to these data with the method of least squares. The results are shown in Fig. 6. The mean of the simulated data lies almost exactly on the fitted straight lines, so the model’s behavior does indeed follow the DT law. This is not a trivial observation because our model derivation is based on a fixed equilibrium criterion and the data points in the plot are computed from the simulated steady-state distribution with a criterion that varies from trial to trial (see Eq. 39 and the explanations at the beginning of Section “Deriving aModel that is Compatible with the DT Law”). Note that for this reason, the slope *a* and the distance $d'$ between the lines that show the fit of the DT law to the simulations in Fig. 6 are also not exactly the same as the *a* and $d'$ parameters in our model that generated those simulated data (cf. Table 2) even though in the model derivation they were the same (and hence have the same name). As the simulated data still follow the DT law, the simulations show that our heuristic simplification that is based on the equilibrium criterion instead of the full equilibrium distribution is a valid simplification for realistic parameter values.

**Table 2:** Parameters of our model fitted to the data from experiment 1 in Stüttgen et al. (2024)

Subject	*a*	$d'$	$\Delta _\text {max}$
1	0.51	5.46	0.72
2	0.27	2.72	1.42
3	0.31	3.62	1.55
4	0.51	3.56	0.96

**Table 3:** BIC values of model fits to the data from experiment 1 in Stüttgen et al. (2024) for the following models: our model introduced in this paper, the income-based KDB model (ib-KDB) from Stüttgen et al. (2013), the trial-based DT model (tb-DT) from Stüttgen et al. (2024)

Subject	Our model	ib-KDB	tb-DT
1	8179	8330	8280
2	14742	15445	15293
3	8335	8794	8495
4	10386	10720	10504

**Fig. 7:**
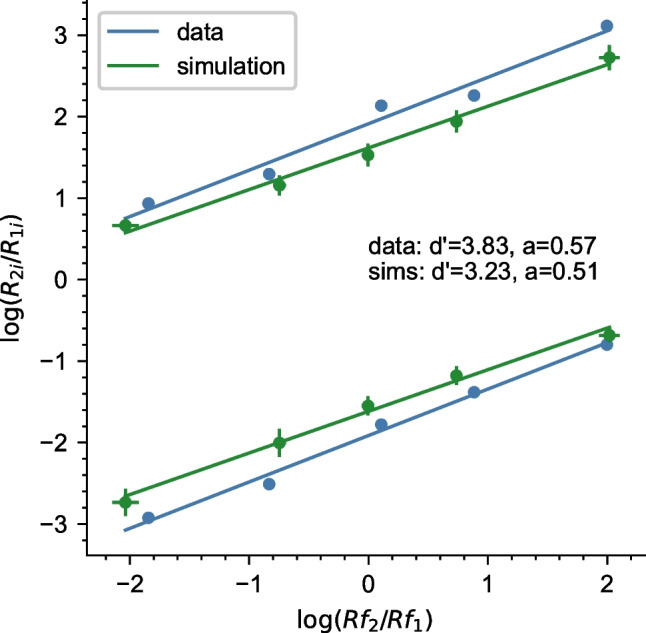
Artificial data was generated by the model with $a=0.5, d'=4, \Delta _\text {max}=0.7$. This plot was produced for the artificial data in the same way that the plots in Fig. 6 were produced for the real experimental data. For each experimental condition, the log response ratio is plotted against the log reinforcement ratio. Blue: artificial data, green: average of 100 simulations of the experiment, simulated with the parameters from the model fit on newly generated stimulus sequences. The horizontal and vertical bars indicate one standard deviation along each axis. Dots represent the data points, the lines result from fitting the DT law with $a_1=a_2 =: a$ and $b^* = 0$ to the data. The parameters of the DT-law fit (a, $d'=2 \log d^*$) are given in the plot

Generally, the experimental data has larger variance between sessions within one condition than the simulations do. This shows that some of the behavioral variability is not accurately captured by our model. This is not terribly surprising because behavioral data are notoriously noisy and there are many factors, such as vigilance, that are hard to control and this additional variance is not modeled here. The sensitivity to reward (slope *a*) that the model simulations display matches well with the empirical sensitivity to reward of the subjects’ behavior. The discriminability (distance $d'$ between the lines) for the model simulations is systematically smaller than the one for the subjects’ behavior. However, this does not imply that the model is a bad description of the behavior. The same phenomenon is observed when fitting the model to data that were generated by simulation of the model (see Fig. 7 in Appendix H) and can therefore be explained by the maximum likelihood estimator being a biased estimator of the true $d^\prime$ underlying the data.

## Discussion

### Theoretical Contributions

Our model brings together three areas of decision-making research that have not yet been unified. It is a trial-by-trial Markovian model that updates an SDT decision criterion and in the steady state, i.e., after learning, displays matching behavior consistent with the DT law. In previously posited criterion learning models (Kac, 1962; Dorfman & Biderman, 1971), the criterion has to be updated after error trials to prevent exclusive choice behavior. Our model does not exhibit this property; there is a steady state in which both responses are emitted probabilistically, while the criterion changes only on rewarded trials.

Our approach rests on an explicit theoretical connection between the DT law and SDT. By deriving the ROC curve, it becomes apparent that behavior that conforms to the DT law can result from choices according to the SDT framework featuring logistic rather than Gaussian stimulus distributions. We also derive the position of the criterion at which such behavior is produced for two different experimental schedules. It can be seen that the DT law implies a direct relationship between a certain experimental condition and the decision criterion an observer adopts in that condition. In designing a trial-by-trial model that learns this criterion position, we derive a new theoretical result about the link between criterion learning models and the DT law: The DT law implies a specific ratio between the update step sizes in either direction in the equilibrium, which directly depends on the criterion position (see Eq. 41).

### Relation to Other Modeling Approaches

Other attempts have been made to combine animal learning theory and SDT to explain perceptual decision-making behavior. In our approach, we start with criterion learning models from SDT and adapt them to be consistent with the DT law. This approach is very much in the tradition of the original work of Davison and Tustin (1978), who have always emphasized the conceptual connections between the DT law and SDT. An obvious alternative to this classic approach is to start with reinforcement learning (RL) models. RL models are well studied in the context of bandit problems and are therefore closely related to Herrnstein’s matching law. Such models often use a logistic response function, also known as a *softmax*, that looks like the generalized matching law (Sutton & Barto, 1998, Section 2.3). Note, however, that in these models, the stochasticity of the responses lies in the decision rule and is not explained by perceptual uncertainty. In fact, it is not immediately obvious how standard RL algorithms, like temporal difference learning or Q-learning, should be adapted to deal with perceptual uncertainty in a way that is psychologically plausible.

Lak et al. (2017, 2020) propose an RL model in which action values are learned for each response option. In each trial, the response with the highest expected value is chosen (they do not use a softmax). These expected values are computed from the learned action values and the probability that each response is the correct one, which is calculated using Gaussian SDT assumptions. Their model can be treated as a trial-by-trial Markovian criterion learning model, even though there is no explicit criterion variable in the model: In each trial, there is an implicit decision criterion whose position depends on the learned values. From trial to trial, these values are updated depending on the perceived stimulus and the received reinforcement, which means that the criterion position changes. In the model by Lak et al. (2020), the update step directly depends on the reward prediction error (RPE), i.e., the difference between the current expected value of the chosen response and the actually received reinforcement. This allows the authors to link behavior to neuronal responses, as they show that the RPE term in their model correlates with the activity of midbrain dopamine neurons that reflect reward prediction errors in classical conditioning experiments (Schultz et al., 1997). A similar approach was taken by Funamizu (2021), who also proposes a model which uses the RPE to learn action values for each response (but without taking the subject’s belief about the observed stimulus into account) and places a decision criterion based on these values.

The learning process in our model differs qualitatively from learning in these RPE-based models: In our model, the criterion is only updated after rewarded trials and remains unchanged after unrewarded trials. In the RPE-based models, the value estimates, and thereby the decision criterion, are updated also when a negative RPE occurs, i.e., when the animal expects a reward but does not receive one. On one hand, this property might make these models more plausible from the theoretical viewpoint of reward prediction. On the other hand, they might not be able to produce steady-state behavior that complies with the DT law. The models are designed in a classical reinforcement learning manner, which aims to learn the optimal behavior and maximize expected rewards. Due to various assumptions in their models, behavior will not necessarily be optimal in the steady state, but, importantly, it is unclear whether and under which assumptions it is consistent with the DT law. Equipping a reinforcement learning model with a probabilistic decision rule, like the softmax, might lead to the desired steady-state behavior. It might also be that the Gaussian noise in the perceptual input leads to behavior that in the steady state is qualitatively similar to the DT law. But we do not know. How to reconcile these models with the empirically well-established DT law is an important theoretical problem that, as far as we can tell, so far has not received the attention in the field of reinforcement learning that it deserves.

In contrast, for models of human criterion updating in signal detection tasks, the steady-state criterion is often studied explicitly and is mostly consistent with probability matching (Dorfman, 1969; Dorfman & Biderman, 1971; Thomas, 1973; Erev, 1998), which is, however, inconsistent with the DT law because in probability matching the response probability only depends on the stimulus presentation probability and is independent of the obtained reinforcements that in turn depend on the response probabilities themselves. To the best of our knowledge, our model is the first trial-by-trial criterion updating model that is consistent with SDT and the DT law (a less worked out precursor to the current model was, however, already published in Stüttgen et al., 2024).

### Limitations and Future Directions

In connecting the DT law to SDT via an ROC curve and criterion, we have posited a fixed criterion position c. This is in line with Davison’s and Tustin’s original interpretation of the DT law, as they draw parallels between their approach and various SDT-like choice measures (Davison & Tustin, 1978). However, in a criterion learning model like ours, the criterion does not actually converge to an equilibrium position; instead, it ends up fluctuating around the equilibrium position in the steady state. Rigorous mathematical treatment of such a steady state (and even showing that a steady-state criterion distribution actually exists) is difficult. While some work has been done to derive this distribution for some versions of the KDB model for small steps as $\Delta$ goes to zero (Norman, 1974), there are no analytical results for larger step sizes. Nevertheless, our model simulations show that the steady-state behavior of our model does follow the DT law closely, even though we only designed the model to follow the DT law for a fixed instead of a fluctuating criterion. Hence, apparently, when applying the model to real data and when the step sizes are small enough, we can approximate the behavior of the subject under the steady-state criterion distribution by a single equilibrium criterion.

The direct link between our model and the DT law is simultaneously its biggest strength and its biggest weakness. By design, it guarantees steady-state behavior in line with a broad range of behavioral findings. On the other hand, the model inevitably inherits some unresolved issues of the DT law. For example, it has been pointed out by Davison and Jenkins (1985) that the sensitivity to reinforcement—*a* in the DT law—has some flaws as a measure. Even within one animal and for the same task, it is inconsistent between different experiments, e.g., an experiment with a URR schedule and an experiment with a CRR schedule (McCarthy & Davison, 1979). Thus, the parameter *a* in our model is specific to each experiment as well and has no generalizable interpretation. Moreover, it is also probably confounded with discriminability (Alsop, 1991; White, 1986). There have been multiple extensions of the original DT law which address these shortcomings (Davison & Jenkins, 1985; Alsop, 1991; Davison, 1991; Davison & Nevin, 1999). Their treatment and relation to the present model is however beyond the scope of the present work and will have to be addressed in the future.

This paper lays the foundation for modelling trial-by-trial decision-making behavior in a way that aligns with the empirical findings about steady-state behavior. It can fit the data from experiment 1 in Stüttgen et al. (2024) better than existing models that try to capture either reinforcement-based criterion-learning or steady-state DT law behavior but do not combine the two. As a next step, our model needs to be evaluated empirically by comparing it to existing models based on other modelling approaches, and in a wider range of scenarios, i.e., on experimental data that systematically varies stimulus probabilities, reinforcement rates, and overall reinforcement density.

So far, in this paper, we only investigated experimental setups with two different stimuli. However, experimental setups with multiple stimuli are used in many experiments (e.g., Davison and McCarthy, 1987; White, 1986; Stüttgen et al., 2011b, 2013; The International Brain Laboratory et al., 2021), and using more than two stimuli confers considerable additional flexibility in experimental design. To adopt our model to these kind of scenarios, further empirical and theoretical research is needed, since the DT law does not hold anymore when stimulus distributions become more complex (Davison & McCarthy, 1987; Davison & Nevin, 1999).

As pointed out in Section “Deriving a Model that is Com-patible with the DT Law,” different step-size functions can be chosen as long as their ratio fulfills (42) in the equilibrium. This allows a modification of the step-size functions to include other factors that are known to be relevant for learning in perceptual decision making. For example, an estimate of the received reinforcement could directly influence the step sizes (cf. Section “Deriving a Model that is Compatiblewith the DT Law”). It would also, in principle, be possible to directly include a dependence on the RPE or some measure of decision confidence. There are some behavioral phenomena that the model in its current form cannot account for, e.g., an overshoot of the response behavior directly after a condition change, as observed in the data in Stüttgen et al. (2024) and Stüttgen et al. (2013). A question for future research is thus whether there are step-size functions with which the model can generate such behavior. Future work should also follow in the footsteps of Treisman and Williams (1984) and look carefully at serial dependencies and check which step-size functions are consistent with them. In general, models with different step-size functions will have to be compared systematically with regard to their theoretical ability to capture relevant phenomena and the goodness of fit to empirical data.

An important open research problem, as already mentioned in Section “Relation to Other Modeling Approaches,” is combining reinforcement learning approaches with the insights about the steady-state criterion presented in this paper. To tackle it, a promising avenue might be to study the partially observable Markov decision process (POMDP) that corresponds to the task. In a POMDP, the agent has to maintain a belief about the (unknown) state of the environment, which should be updated after each observation in a Bayesian manner. Decisions are then made based on the current belief. In our task, subjects should therefore maintain a belief about the probability that a certain action will lead to a reinforcement. Like in standard reinforcement learning models that deal with fully observable Markov decision processes, the assumption is that an agent in a POMDP should try to maximize the expected reward, i.e., the goal for the agent is to behave optimally. Still, the approach might be capable of producing DT law behavior, which is suboptimal (but see Sakai and Fukai, 2008). Suboptimal behavior can arise from a non-deterministic decision rule or from the way the belief distribution is maintained. For example, it has been suggested by Mozer et al. (2008) that humans store a limited number of samples rather than a full probability distribution. And Vul et al. (2014) show that such a sampling strategy can give rise to matching-law behavior in two-armed bandit tasks. In a similar way, it is conceivable that approximate Bayesian approaches might lead to behavior in line with the DT law in a signal-detection task, given the right assumptions.

## Conclusion

Research on learning in perceptual decision making takes many forms. Some research is firmly based in signal detection theory and provides trial-by-trial Markovian models of criterion updating. Other research focuses on behavioral regularities in the steady state, like the DT law. Reinforcement learning approaches take into account biologically plausible components like the reward prediction error. All these approaches provide valuable insights into decision behavior and the process by which it is learned. However, a unified account is still missing.

We have highlighted the theoretical connections between some of these approaches. Moreover, we have showcased how a model can be designed to link the different aspects of perceptual decision making to each other. The result is a model grounded in SDT with a criterion that is updated from trial to trial. In this model, the size of the update steps depends on the current criterion position and yields behavior that follows the DT law in the steady state. The update steps towards even more extreme criteria get smaller the more extreme the criterion already is. This mechanism allows the model to learn only from reinforced responses while still being stable, unlike the original KDB reward-learning model. Our work hence provides a theoretical as well as practical link between SDT, trial-by-trial learning models, and descriptions of steady-state behavior.

## Data Availability

The data from experiment 1 in Stüttgen et al. (2024) that were analyzed in this paper are available in the Open Science Framework repository at https://osf.io/y8xek/.

## Appendix A: Reason for Reparameterizing the DT Law

We use a different parameterization than the one that is usually used for the DT law because the original parameterization confounds bias and discriminability in the unequal-variance case. To demonstrate that, let us look at what happens when we derive the ROC curve using the DT law with the original discriminability and bias measures (Eqs. 7 and 8). This derivation works exactly as the derivation with our new measures, which is done in Eqs. 13, 14, 15, 16, 17, 18 and 19. Expressing the DT law in terms of log-odds of hit rate and false alarm rate gives

A1 $$\begin{aligned} \sigma ^{-1}(f)&= a_1 \log \left( \frac{Rf_2}{Rf_1} \right) + \log b - \log d \end{aligned}$$

A2 $$\begin{aligned} \sigma ^{-1}(h)&= a_2 \log \left( \frac{Rf_2}{Rf_1} \right) + \log b + \log d. \end{aligned}$$

Rearranging these terms leads to

A3 $$\begin{aligned} \frac{1}{a_1} \left[ \sigma ^{-1}(f) - \log b + \log d \right]&= \log \left( \frac{Rf_2}{Rf_1} \right) \end{aligned}$$

A4 $$\begin{aligned} \frac{1}{a_2} \left[ \sigma ^{-1}(h) - \log b - \log d \right]&= \log \left( \frac{Rf_2}{Rf_1} \right) . \end{aligned}$$

For both equations to hold simultaneously, we need

A5 $$\begin{aligned}&\frac{1}{a_1} \left[ \sigma ^{-1}(f) - \log b + \log d \right] = \frac{1}{a_2} \left[ \sigma ^{-1}(h) - \log b - \log d \right] \end{aligned}$$

A6 $$\begin{aligned} \Leftrightarrow&\frac{1}{a_2} \sigma ^{-1}(h) = \frac{1}{a_1} \sigma ^{-1}(f) + \left( \frac{1}{a_2} + \frac{1}{a_1} \right) \log d + \left( \frac{1}{a_2} - \frac{1}{a_1} \right) \log b \end{aligned}$$

A7 $$\begin{aligned} \Leftrightarrow&\sigma ^{-1}(h) = \frac{a_2}{a_1} \sigma ^{-1}(f) + \left( 1 + \frac{a_2}{a_1} \right) \log d + \left( 1 - \frac{a_2}{a_1} \right) \log b. \end{aligned}$$

This ROC curve corresponds to logistic stimulus distributions with scales $s_1=\frac{a_2}{a_1}$, $s_2=1$ and

A8 $$\begin{aligned} d' = \left( 1 + \frac{a_2}{a_1} \right) \log d + \left( 1 - \frac{a_2}{a_1} \right) \log b. \end{aligned}$$

$d'$ is the SDT measure for how discriminable the two stimuli are. Therefore, if *d* and *b* are measures of discriminability and bias, respectively, there should be a one-to-one correspondence from *d* to $d'$ which does not depend on *b*. However, here, *d* and *b* are confounded. Only in the equal-variance case ($a_1=a_2$ and therefore $s_1=1=s_2$) $d'$ becomes independent of *b* and is a valid bias-independent measure of discriminability.

## Appendix B: SDT with Logistic Stimulus Distributions

Consider two logistic stimulus distributions

B9 $$\begin{aligned} p(x|S=i) = \frac{e^{-\frac{x-\mu _i}{s_i}}}{1 + e^{-\frac{x-\mu _i}{s_i}}} = \mathcal {L}\left( x; \mu _i, s_i \right) . \end{aligned}$$

The hit rate and false alarm rate can be computed as follows:

B10 $$\begin{aligned} h&= 1 - \int _{-\infty }^c \mathcal {L}\left( x; \mu _2, s_2 \right) = 1 - \sigma \left( \frac{c-\mu _2}{s_2} \right) = \sigma \left( \frac{-c+\mu _2}{s_2} \right) \end{aligned}$$

B11 $$\begin{aligned} f&= 1 - \int _{-\infty }^c \mathcal {L}\left( x; \mu _1, s_1 \right) = 1 - \sigma \left( \frac{c-\mu _1}{s_1} \right) = \sigma \left( \frac{-c+\mu _1}{s_1}\right) . \end{aligned}$$

### B.1 Derivation of ROC Curve

From Eqs. B10 and B11, it follows that

B12 $$\begin{aligned} \sigma ^{-1}(h)&= \frac{-c+\mu _2}{s_2} \end{aligned}$$

B13 $$\begin{aligned}&= \frac{-c+\mu _1}{s_2} + \frac{\mu _2-\mu _1}{s_2} \end{aligned}$$

B14 $$\begin{aligned}&= \frac{s_1}{s_2} \frac{-c+\mu _1}{s_1} + \frac{\mu _2-\mu _1}{s_2} \end{aligned}$$

B15 $$\begin{aligned}&= \frac{s_1}{s_2} \sigma ^{-1}(f) + \frac{\mu _2-\mu _1}{s_2}. \end{aligned}$$

For $s_2 = s$, $s_1 = \frac{a_2}{a_1} s$ and $d^{\prime }:= \mu _2-\mu _1 = s_2 \left( 1+\frac{a_2}{a_1}\right)$ $\log d^* = (s_1+s_2) \log d^*$, this is the ROC curve derived from the DT law (see Eq. 19). In particular, for $s_2 = 1$, we get $s_1 = \frac{a_2}{a_1}$ and $d^{\prime } = \left( 1+\frac{a_2}{a_1}\right) \log d^*$.

### B.2 Criterion Corresponding to DT Law

For logistic stimulus distributions with means $\mu _1=-\frac{d'}{2}$, $\mu _2=\frac{d'}{2}$ and scales $s_1=\frac{a_2}{a_1}$, $s_2=1$, hit rate and false alarm rate are

B16 $$\begin{aligned} h&= \sigma \left( -c+\frac{d'}{2} \right) \end{aligned}$$

B17 $$\begin{aligned} f&= \sigma \left( \left( -c-\frac{d'}{2}\right) \frac{a_1}{a_2} \right) \end{aligned}$$

Therefore, the criterion *c* can be computed from the reinforcement ratios as

B18 $$\begin{aligned} c&= - \frac{1}{2} \left[ \sigma ^{-1}(h) + \frac{a_2}{a_1} \sigma ^{-1}(f))\right] \end{aligned}$$

B19 $$\begin{aligned}&= -\frac{1}{2} \left[ \log \left( \frac{R_{22}}{R_{12}} \right) + \frac{a_2}{a_1} \log \left( \frac{R_{21}}{R_{11}} \right) \right] \end{aligned}$$

B20 $$\begin{aligned}&= - \frac{1}{2} \Bigg [a_2 \log \!\left( \frac{Rf_{2}}{Rf_{1}}\right) \! +\! a_2 \log b^* \!+\! \log d^* + a_2 \log \!\left( \frac{Rf_{2}}{Rf_{1}}\right) \nonumber \\&\quad + a_2 \log b^* - \frac{a_2}{a_1} \log d^*\Bigg ] \end{aligned}$$

B21 $$\begin{aligned}&= - a_2 \log \left( \frac{Rf_{2}}{Rf_{1}} \right) - a_2 \log b^* - \frac{1}{2} \left( 1 - \frac{a_2}{a_1} \right) \log d^* \end{aligned}$$

B22 $$\begin{aligned}&= - a_2 \log \left( \frac{Rf_2}{Rf_1} b^* \right) - \frac{1}{2} \left( 1 - \frac{a_2}{a_1} \right) \frac{d'}{1+\frac{a_2}{a_1}} \end{aligned}$$

B23 $$\begin{aligned}&= - a_2 \log \left( \frac{Rf_2}{Rf_1} b^* \right) - \frac{1}{2} \left( \frac{a_1-a_2}{a_1} \right) \frac{d'}{\frac{a_1+a_2}{a_1}} \end{aligned}$$

B24 $$\begin{aligned}&= - a_2 \log \left( \frac{Rf_2}{Rf_1} b^* \right) - \frac{1}{2} \left( \frac{a_1-a_2}{a_1+a_2} \right) d' \end{aligned}$$

B25 $$\begin{aligned}&= - a_2 \log \left( \frac{Rf_2}{Rf_1} b^* \right) + c_0 \end{aligned}$$

with

B26 $$\begin{aligned} c_0 := - \frac{1}{2} \frac{a_1-a_2}{a_1+a_2} d'. \end{aligned}$$

For the uncontrolled reinforcement schedule, we get

B27 $$\begin{aligned} c&= - a_2 \log \left( \frac{Rf_{2}}{Rf_{1}} b^* \right) + c_0 \end{aligned}$$

B28 $$\begin{aligned}&= - a_2 \log \left( \frac{R_{22}}{R_{11}} \frac{\pi _2 r_2}{\pi _1 r_1} b^* \right) + c_0 \end{aligned}$$

B29 $$\begin{aligned}&= - a_2 \log \left( \frac{\sigma \left( d'/2-c\right) }{\sigma \left( a_1/a_2\left( d'/2+c\right) \right) } \frac{\pi _2 r_2}{\pi _1 r_1} b^* \right) + c_0\end{aligned}$$

B30 $$\begin{aligned}&= - a_2 \log \left( \frac{1+e^{-a_1/a_2 \left( d'/2+c\right) }}{1+e^{-\left( d'/2-c\right) }} \frac{\pi _2 r_2}{\pi _1 r_1} b^* \right) + c_0. \end{aligned}$$

### B.3 Neutral Criterion

The neutral criterion is the value $c_0$ where the two stimulus distributions intersect, i.e., $p(x=c_0|S=1) = p(x=c_0|S=2)$. For logistic distributions with $\mu _1=-\frac{d'}{2}$, $\mu _2=\frac{d'}{2}$, $s_1=\frac{a_2}{a_1}$, $s_2=1$, the neutral criterion is

B31 $$\begin{aligned} c_0 := - \frac{1}{2} \frac{a_1-a_2}{a_1+a_2} d'. \end{aligned}$$

#### Proof

Let us denote

B32 $$\begin{aligned} f(x) = \mathcal {L}(x;0,1) = \frac{e^{-x}}{\left( 1 + e^{-x}\right) ^2}. \end{aligned}$$

Note that this is a symmetric function, i.e., $f(x) = f(-x)$. Plugging $x=c_0$ into the stimulus distribution function for the first stimulus

B33 $$\begin{aligned} p(c_0|S=1)&= \mathcal {L}\left( c_0; -\frac{d'}{2}, \frac{a_2}{a_1} \right) \end{aligned}$$

B34 $$\begin{aligned}&= f\left( \frac{a_1}{a_2}\left( c_0+\frac{d'}{2}\right) \right) \end{aligned}$$

B35 $$\begin{aligned}&= f\left( \frac{a_1}{a_2}\left( - \frac{1}{2} \frac{a_1-a_2}{a_1+a_2} d'+d'/2\right) \right) \end{aligned}$$

B36 $$\begin{aligned}&= f\left( \frac{d'}{2} \frac{a_1}{a_2} \left( 1 - \frac{a_1-a_2}{a_1+a_2}\right) \right) \end{aligned}$$

B37 $$\begin{aligned}&= f\left( \frac{d'}{2} \frac{a_1}{a_2} \frac{a_1+a_2-(a_1-a_2)}{a_1+a_2}\right) \end{aligned}$$

B38 $$\begin{aligned}&= f\left( \frac{d'}{2} \frac{a_1}{a_2} \frac{2 a_2}{a_1+a_2}\right) \end{aligned}$$

B39 $$\begin{aligned}&= f\left( \frac{a_1}{a_1+a_2} d'\right) \end{aligned}$$

and the second stimulus

B40 $$\begin{aligned} p(c_0|S=2)&= \mathcal {L}\left( c_0; \frac{d'}{2}, 1 \right) \end{aligned}$$

B41 $$\begin{aligned}&= f\left( c_0-d'/2\right) \end{aligned}$$

B42 $$\begin{aligned}&= f\left( - \frac{1}{2} \frac{a_1-a_2}{a_1+a_2} d'-d'/2\right) \end{aligned}$$

B43 $$\begin{aligned}&= f\left( \frac{d'}{2} \left( -1 - \frac{a_1-a_2}{a_1+a_2}\right) \right) \end{aligned}$$

B44 $$\begin{aligned}&= f\left( \frac{d'}{2} \frac{-(a_1+a_2)-(a_1-a_2)}{a_1+a_2}\right) \end{aligned}$$

B45 $$\begin{aligned}&= f\left( \frac{d'}{2} \frac{-2 a_1}{a_1+a_2}\right) \end{aligned}$$

B46 $$\begin{aligned}&= f\left( -\frac{a_1}{a_1+a_2} d'\right) \end{aligned}$$

B47 $$\begin{aligned}&= f\left( \frac{a_1}{a_1+a_2} d'\right) \end{aligned}$$

shows that $p(c_0|S=1) = p(c_0|S=2)$ at the neutral criterion $c_0$, i.e., the two distributions intersect.

## Appendix C: Proof of Existence of a DT Criterion

For a URR schedule, we derived the fixed-point equation (27) for the criterion position. We can rewrite this as

C48 $$\begin{aligned}&c=a_2 \log \left( 1+e^{-\left( d^{\prime } / 2-c\right) }\right) -a_2 \log \left( 1+e^{-a_1/a_2(d^{\prime } / 2+c)}\right) +y \end{aligned}$$

C49 $$\begin{aligned} \Leftrightarrow&y \!=\! c-a_2 \log \left( 1+e^{-(d^{\prime } / 2-c)}\right) \!+a_2 \log \left( 1\!+\!e^{-a_1/a_2(d^{\prime } / 2+c)}\right) \!=: g(c), \end{aligned}$$

with

C50 $$\begin{aligned} y = - a_2 \log \left( \frac{\pi _2 r_2}{\pi _1 r_1} b^* \right) + c_0. \end{aligned}$$

*y* can take any value in $\mathbb {R}$ depending on the condition. So to show that for every condition there exists a criterion which will lead to DT-law behavior, we need to show that for every $y\in \mathbb {R}$, there is a *c* fulfilling Eq. C49, i.e., that the value range of *g*(*c*) is $\mathbb {R}$. Since *g*(*c*) is continuous, it suffices to look at the limits for $c \rightarrow \infty$ and $c \rightarrow -\infty$.

For large positive *c*,

C51 $$\begin{aligned} \log \left( 1+e^{-\left( \frac{d^{\prime }}{2}-c\right) }\right) \approx \log \left( e^{-\left( \frac{d^{\prime }}{2}-c\right) }\right) =c-\frac{d^{\prime }}{2} \end{aligned}$$

and

C52 $$\begin{aligned} \lim _{c \rightarrow \infty } \log \left( 1+e^{-a_1/a_2\left( \frac{d^{\prime }}{2}+c\right) }\right) =0, \end{aligned}$$

so we get

C53 $$\begin{aligned} g(c) \approx c-a_2\left( c-\frac{d^{\prime }}{2}\right) =(1-a_2) c+a_2 \frac{d^{\prime }}{2}. \end{aligned}$$

Similarly, for large negative c,

C54 $$\begin{aligned} \lim _{c \rightarrow \infty } \log \left( 1+e^{-\left( \frac{d^{\prime }}{2}-c\right) }\right) =0 \end{aligned}$$

and

C55 $$\begin{aligned} \log \left( 1+e^{-a_1/a_2\left( \frac{d^{\prime }}{2}+c\right) }\right) \approx \log \left( e^{-a_1/a_2\left( \frac{d^{\prime }}{2}+c\right) }\right) =\frac{a_1}{a_2}\left( -c-\frac{d^{\prime }}{2}\right) , \end{aligned}$$

so we get

C56 $$\begin{aligned} g(c) \approx c+a_1\left( -c-\frac{d^\prime }{2}\right) =(1-a_1) c-a_1 \frac{d^{\prime }}{2}. \end{aligned}$$

That is, for $0<a_1<1$ and $0<a_2<1$, *g*(*c*) increases linearly both for $c \rightarrow \infty$ and $c \rightarrow -\infty$, thereby having a value range of $\mathbb {R}$. That proves that there is a DT criterion for any condition in the case of undermatching.

For $a_1=1$ (or $a_2=1$), one of the limits becomes constant at $\lim _{c \rightarrow -\infty } g(c) = -d'/2$ (or $\lim _{c \rightarrow -\infty } g(c) = d'/2$, respectively). That means a DT criterion exists for conditions with $y>-d'/2$ (or $y<d'/2$), with $c \rightarrow -\infty$ for $y \rightarrow -d'/2$ (or $c \rightarrow \infty$ for $y \rightarrow d'/2$). For reinforcement rates leading to more extreme values of *y*, behavior that is consistent with the DT law can be generated by exclusively choosing the response option that is favored by the ratio.

A similar but slightly more complicated analysis can be done for $a>1$. We limit the investigations in this paper to the case of $a \le 1$ though, as overmatching behavior is usually not observed empirically (see, e.g., Baum, 1979).

## Appendix D: Step-Size Function

To keep step sizes bounded, we want

D57 $$\begin{aligned} \Delta _{11}(c) + \Delta _{22}(c) = 1 \end{aligned}$$

for any *c*. Moreover, the step-size functions need to fulfill the ratio constraint from Eq. 42, so

D58 $$\begin{aligned} \Delta _{11}(c) / \Delta _{22}(c) = e^{-c/a}. \end{aligned}$$

By solving Eq. D57 for $\Delta _{22}(c)$ and plugging it into Eq. D58, we find

D59 $$\begin{aligned} &  \Delta _{11}(c) / (1-\Delta _{11}(c))&= e^{-c/a} \end{aligned}$$

D60 $$\begin{aligned} \Leftrightarrow &  \Delta _{11}(c)&= (1-\Delta _{11}(c)) e^{-c/a} \end{aligned}$$

D61 $$\begin{aligned} \Leftrightarrow &  \Delta _{11}(c) (1+e^{-c/a})&= e^{-c/a} \end{aligned}$$

D62 $$\begin{aligned} \Leftrightarrow &  \Delta _{11}(c)&= e^{-c/a} / (1+e^{-c/a}) \end{aligned}$$

and accordingly

D63 $$\begin{aligned} \Delta _{22}(c) = 1-\Delta _{11}(c) = 1 / (1+e^{-c/a}). \end{aligned}$$

Note that this can be rewritten as

D64 $$\begin{aligned} \Delta _{22}(c) = e^{c/a} / (e^{c/a}+1) = \Delta _{11}(-c), \end{aligned}$$

so the step-size functions are symmetric to each other, fulfilling our other desired constraint.

## Appendix E: Model with Step Sizes Dependent on Reinforcements

Recall that a KDB-like criterion updating model with step-size functions $\Delta _{11}(c)$ and $\Delta _{22}$ is in an equilibrium at criterion $\hat{c}$ when

$$\begin{aligned} \frac{\Delta _{11}(\hat{c})}{\Delta _{22}(\hat{c})} = \frac{Rf_2(\hat{c})}{Rf_1(\hat{c})} \end{aligned}$$

(see Eq. 38), and moreover that the DT criterion, i.e., the criterion that is consistent with the DT law, is given by

$$\begin{aligned} c = - a \log \left( \frac{Rf_2}{Rf_1} b^* \right) \end{aligned}$$

(see Eq. 39).

In the main text of this paper, we have derived a possible step-size ratio $\Delta _{11}(c)/\Delta _{22}(c)$ for which the equilibrium $\hat{c}$ is the DT criterion. We have done so by solving Eq. 39 for $Rf_{2}/Rf_{1}$, which gives

$$\begin{aligned} \frac{Rf_2}{Rf_1} = \frac{1}{b^*} e^{-\hat{c}/a} \end{aligned}$$

(see Eq. 40), and plugging this into the right-hand side of Eq. 38.

There are other ways to derive a step-size ratio for which the equilibrium $\hat{c}$ is the DT criterion, too. For example, it could be desirable to have step-size functions that depend directly on the received reinforcements. To achieve that, a recursive formulation of $Rf_{2}/Rf_{1}$ is needed, which can, e.g., be derived by squaring Eq. 40:

E65 $$\begin{aligned}&\left( \frac{Rf_2}{Rf_1}\right) ^2 = \left( \frac{1}{b^*}\right) ^2 e^{-2c/a} \end{aligned}$$

E66 $$\begin{aligned} \Leftrightarrow&\frac{Rf_2}{Rf_1} = \left( \frac{1}{b^*}\right) ^2 e^{-2c/a} \frac{Rf_1}{Rf_2} \end{aligned}$$

This can then be plugged into Eq. 38 as before. Again imposing the additional constraints and assuming an unbiased observer, step-size functions can, e.g., be chosen as

E67 $$\begin{aligned} \Delta _{11}(c)&= \Delta _\text {max} \frac{1}{1+e^{2c/a}} Rf_1(c)\end{aligned}$$

E68 $$\begin{aligned} \Delta _{22}(c)&= \Delta _\text {max} \frac{1}{1+e^{-2c/a}} Rf_2(c) \end{aligned}$$

Since a subject cannot directly know the expected reinforcement $Rf_i$ for each response at its current criterion position, an estimate based on previously received reinforcements has to be used instead.

## Appendix F: Existence and Stability of Model Equilibrium

We are going to show that for any condition in a URR schedule, there is exactly one criterion position $\hat{c}$ where our model (with $a<1$) is in an equilibrium, and moreover, that this equilibrium is stable. For a model with $a\ge 1$, a similar proof is possible, but this is left out of this paper, as we are not interested in models that display overmatching behavior.

The expected update step of a KDB-like model is

F69 $$\begin{aligned} \mathbb {E}(\Delta |c) = Rf_1(c)\Delta _{11}(c) - Rf_2(c) \Delta _{22}(c). \end{aligned}$$

The model is in an equilibrium at $\hat{c}$ if and only if

F70 $$\begin{aligned} &  \mathbb {E}(\Delta |\hat{c})&= 0 \end{aligned}$$

F71 $$\begin{aligned} \Leftrightarrow &  Rf_1(\hat{c})\Delta _{11}(\hat{c})&= Rf_2(\hat{c}) \Delta _{22}(\hat{c}) \end{aligned}$$

F72 $$\begin{aligned} \Leftrightarrow &  \log \left( Rf_1(\hat{c})\Delta _{11}(\hat{c}) \right)&= \log \left( Rf_2(\hat{c})\Delta _{22}(\hat{c})\right) \end{aligned}$$

F73 $$\begin{aligned} \Leftrightarrow &  \log \left( Rf_1(\hat{c})\Delta _{11}(\hat{c}) \right) - \log \left( Rf_2(\hat{c})\Delta _{22}(\hat{c})\right)&= 0 \end{aligned}$$

Let us call

F74 $$\begin{aligned} g(c) := \log \left( Rf_1(c)\Delta _{11}(c) \right) - \log \left( Rf_2(c)\Delta _{22}(c)\right) . \end{aligned}$$

Plugging the step sizes of our model (Eqs. 43 and 44) and the expected reinforcements for a URR schedule in, we get

F75 $$\begin{aligned} g(c) =&\log \left( \pi _1 r_1 \sigma \left( c+d^{\prime }/2\right) \frac{\Delta _\text {max}}{1+e^{c/a}} \right) \nonumber \\&\quad - \log \left( \pi _2 r_2 \sigma \left( -c+d^{\prime }/2\right) \frac{\Delta _\text {max}}{1+e^{-c/a}} \right) \end{aligned}$$

F76 $$\begin{aligned} =&\log \left( \pi _1 r_1 \frac{1}{1+e^{-c-d^{\prime }/ 2}} \frac{\Delta _\text {max}}{1+e^{c/a}}\right) \nonumber \\&\quad - \log \left( \pi _2 r_2 \frac{1}{1+e^{c-d^{\prime }/2}} \frac{\Delta _\text {max}}{1+e^{-c/a}} \right) \end{aligned}$$

F77 $$\begin{aligned} =&\log \left( \pi _1 r_1 \right) - \log \left( 1+e^{-c-d^{\prime }/ 2} \right) - \log \left( 1+e^{c/a} \right) \end{aligned}$$

F78 $$\begin{aligned}&- \log \left( \pi _2 r_2 \right) + \log \left( 1+e^{c-d^{\prime }/2} \right) + \log \left( 1+e^{-c/a}\right) \end{aligned}$$

The equilibrium is stable if $\mathbb {E}(\Delta |c) > 0$ for $c<\hat{c}$ and $\mathbb {E}(\Delta |c) < 0$ for $c>\hat{c}$. In analogy to the previous calculation for the equilibrium criterion, this is equivalent to $g(c) > 0$ for $c<\hat{c}$ and $g(c) < 0$ for $c>\hat{c}$.

We are now going to show that

1. *g*(*c*) is monotonically decreasing with
2. $$\lim _{c\rightarrow \infty }g(c) = -\infty $$ and
3. $$\lim _{c\rightarrow -\infty }g(c) = \infty $$.

Together, this proves that *g*(*c*) has exactly one zero crossing $\hat{c}$ with $g(c) > 0$ for $c<\hat{c}$ and $g(c) < 0$ for $c>\hat{c}$, which means that our model has exactly one equilibrium and this equilibrium is stable.

### Proof of 1

The derivative of *g*(*c*) with respect to *c* is

F79 $$\begin{aligned} g'(c)&= \frac{e^{c-d'/2}}{1+e^{c-d'/2}} + \frac{-\frac{1}{a}e^{-c/a}}{1+e^{-c/a}} - \frac{-e^{-c-d'/2}}{1+e^{-c-d'/2}} - \frac{\frac{1}{a}e^{c/a}}{1+e^{c/a}} \end{aligned}$$

F80 $$\begin{aligned}&= \frac{e^{c-d'/2}}{1+e^{c-d'/2}} - \frac{1}{a} \frac{e^{-c/a}}{1+e^{-c/a}} + \frac{1}{1+e^{c+d'/2}} - \frac{1}{a} \frac{1}{1+e^{-c/a}} \end{aligned}$$

F81 $$\begin{aligned}&= \frac{e^{c-d'/2}}{1+e^{c-d'/2}} + \frac{1}{1+e^{c+d'/2}} - \frac{1}{a} \frac{1+e^{-c/a}}{1+e^{-c/a}} \end{aligned}$$

F82 $$\begin{aligned}&= \frac{e^{c-d'/2}}{1+e^{c-d'/2}} + \frac{1}{1+e^{c+d'/2}} - \frac{1}{a}. \end{aligned}$$

Since $d'>0$, $e^{c+d'/2}>e^{c-d'/2}$ and therefore

F83 $$\begin{aligned} g'(c) < \frac{e^{c-d'/2}}{1+e^{c-d'/2}} + \frac{1}{1+e^{c-d'/2}} - \frac{1}{a} = 1-\frac{1}{a}. \end{aligned}$$

For $a<1$ (undermatching), this is smaller than zero. A negative first derivative for every *c* means that the function *g*(*c*) is monotonically decreasing.

### Proof of 2

For large positive *c*,

F84 $$\begin{aligned}&\lim _{c \rightarrow \infty } \log \left( 1+e^{-c-d'/2}\right) =0, \end{aligned}$$

F85 $$\begin{aligned}&\log \left( 1+e^{c/a}\right) \approx \log \left( e^{c/a}\right) =\frac{c}{a}, \end{aligned}$$

F86 $$\begin{aligned}&\log \left( 1+e^{c-d'/2}\right) \approx \log \left( e^{c-d'/2}\right) =c-\frac{d^{\prime }}{2}, \text { and} \end{aligned}$$

F87 $$\begin{aligned}&\lim _{c \rightarrow \infty } \log \left( 1+e^{-c/a}\right) =0. \end{aligned}$$

Putting everything together, we get

F88 $$\begin{aligned} g(c)&\approx \log \left( \pi _1 r_1 \right) - \frac{c}{a} - \log \left( \pi _2 r_2 \right) + c-\frac{d^{\prime }}{2} \end{aligned}$$

F89 $$\begin{aligned}&= \left( 1 - \frac{1}{a}\right) c + \log \left( \frac{\pi _1r_1}{\pi _2r_2}\right) - \frac{d^{\prime }}{2}. \end{aligned}$$

For $a<1$, this is linear with a negative slope, so $\lim _{c\rightarrow \infty } g(c) = -\infty$.

### Proof of 3

For large negative *c*,

F90 $$\begin{aligned}&\log \left( 1+e^{-c-d'/2}\right) \approx \log \left( e^{-c-d'/2}\right) = -c - \frac{d^{\prime }}{2}, \end{aligned}$$

F91 $$\begin{aligned}&\lim _{c \rightarrow -\infty } \log \left( 1+e^{c/a}\right) = 0, \end{aligned}$$

F92 $$\begin{aligned}&\lim _{c \rightarrow -\infty } \log \left( 1+e^{c-d'/2}\right) = 0, \text { and} \end{aligned}$$

F93 $$\begin{aligned}&\log \left( 1+e^{-c/a}\right) \approx \log \left( e^{-c/a}\right) =-\frac{c}{a}. \end{aligned}$$

Putting everything together, we get

F94 $$\begin{aligned} g(c)&\approx \log \left( \pi _1 r_1 \right) - \left( -c - \frac{d^{\prime }}{2} \right) - \log \left( \pi _2 r_2 \right) -\frac{c}{a} \end{aligned}$$

F95 $$\begin{aligned}&= \left( 1 - \frac{1}{a}\right) c + \log \left( \frac{\pi _1r_1}{\pi _2r_2}\right) + \frac{d^{\prime }}{2}. \end{aligned}$$

For $a<1$, this is again linear with a negative slope, so $\lim _{c\rightarrow \infty } g(c) = \infty$.

## Appendix G: Leaky KDB Model Incompatible with DT Law

Stüttgen et al. (2013) derive that there is a linear relationship between the equilibrium criterion of their model and the (weighted) difference between the expected reinforcement for response 1 and response 2. For a model with step sizes $\Delta _{11}$, $\Delta _{22}$ and leak-term $\gamma$,

G96 $$\begin{aligned} c = \frac{1}{1-\gamma } \left( \Delta _{11}Rf_1 - \Delta _{22}Rf_2 \right) . \end{aligned}$$

In contrast, the DT criterion depends linearly on the difference between the logarithms of the expected reinforcements:

G97 $$\begin{aligned} c = a_2 \left( \log ({Rf_1}) - \log ({Rf_2}) \right) - a_2 \log (b^*) + c_0 \end{aligned}$$

(cf. Eq. 25), with the last two additive terms being equal to zero for an unbiased observer with symmetric sensitivities to reward. These functional relationships are fundamentally different from each other, so the model is not compatible with the DT law, no matter the choice of parameters.

## Code Availibility

The code is available at https://osf.io/y8xek/.
